# i5hmCVec: Identifying 5-Hydroxymethylcytosine Sites of *Drosophila* RNA Using Sequence Feature Embeddings

**DOI:** 10.3389/fgene.2022.896925

**Published:** 2022-05-03

**Authors:** Hang-Yu Liu, Pu-Feng Du

**Affiliations:** College of Intelligence and Computing, Tianjin University, Tianjin, China

**Keywords:** 5-hydroxymethylcytosine, dna2vec, machine learning, cross-validation, i5hmcVec

## Abstract

5-Hydroxymethylcytosine (5hmC), one of the most important RNA modifications, plays an important role in many biological processes. Accurately identifying RNA modification sites helps understand the function of RNA modification. In this work, we propose a computational method for identifying 5hmC-modified regions using machine learning algorithms. We applied a sequence feature embedding method based on the dna2vec algorithm to represent the RNA sequence. The results showed that the performance of our model is better that of than state-of-art methods. All dataset and source codes used in this study are available at: https://github.com/liu-h-y/5hmC_model.

## Introduction

Posttranscriptional modifications have been extensively studied over the last few years. More than 160 types of modification have been identified across all kingdoms of life (Boccaletto et al., 2018). Posttranscriptional modifications play important roles in various biological processes, such as RNA degradation (Sommer et al., 1978), RNA splicing (Lindstrom et al., 2003), and transcriptional regulations (Cowling, 2009). To understand the mechanism of RNA modifications, it is important to pinpoint the modification sites in the RNA sequences (Dominissini et al., 2012; Meyer et al., 2012).

With the rapid development of high-throughput technology, several experimental methods for identifying RNA modification sites have been developed, such as MERIP (Meyer et al., 2012) and m6A-seq (Dominissini et al., 2012). These methods are more capable of picking up the modified transcripts or regions on the transcripts, rather than accurately pinpointing the modification sites. With the advances in modern life sciences, especially the cross-linking technology, methods for identifying RNA modification sites at single-base resolution were also proposed, including miCLIP (Linder et al., 2015), PA-m6A-seq (Kai Chen et al., 2015), and m7G-MeRIP-seq (Zhang et al., 2019). However, these experimental methods are still costly and time-consuming. Therefore, computational methods have been proposed as alternative approaches. A series of bioinformatics tools using machine learning algorithms for predicting m6A (Wei Chen et al., 2015; Zhou et al., 2016; Huang et al., 2018; Kunqi Chen et al., 2019; Zou et al., 2019), m5C (Qiu et al., 2017; Sabooh et al., 2018; Akbar et al., 2020; Dou et al., 2020), m7G (Wei Chen et al., 2019, 7; Liu X. et al., 2020; Yang et al., 2020; Dai et al., 2021), and many others have been developed. A recent review article has elaborated on the differences between these studies, in the aspect of benchmarking datasets, feature encoding schemes, and the main algorithms (Chen et al., 2020).

5-Hydroxymethylcytosine (5hmC) plays a key role in various cellular processes. 5hmC modification exists on both RNA and DNA sequences (Zhang et al., 2016). Most of the existing studies focused on the DNA 5hmC modifications (Szwagierczak et al., 2010; Pastor et al., 2011; Yu et al., 2012; Bachman et al., 2014). The RNA 5hmC modifications were much less studied (Fu et al., 2014; Huber et al., 2015; Delatte et al., 2016; Miao et al., 2016). Fu et al. first found that the m5C site can be catalyzed by the Tet enzyme to form 5hmC sites with a ratio of about 0.02% *in vitro* in mammalian RNA (Fu et al., 2014). In addition, a discovery that Tet-mediated oxidation of m5C in RNA is much less efficient than that in DNA (Fu et al., 2014). Huber et al. verified that 5hmC is the result of m5C oxidation *in vivo* in a mouse model using an isotope-tracing methodology (Huber et al., 2015). They also found that in worms and plants, the formation of 5hmC in RNA does not require a Tet-mediated oxidation mechanism. Miao et al. (2016) found that 5hmC in RNA is rich in the mouse brain, which is potentially related to brain functions. Delatte et al. (2016) systematically identified 5hmC modifications in *Drosophila* transcriptome using the hMeRIP-seq method. Using the data from Delatte et al., Liu et al. developed a predictor iRNA5hmC for computationally identifying 5hmC modifications with machine learning algorithms (Liu Y. et al., 2020). Ahmed et al. also constructed a predictor iRNA5hmC-PS (Ahmed et al., 2020) by using position-specific binary indicators of RNA sequences. However, Delatte et al. did not provide the exact location of 5hmC modification sites in the transcriptome (Delatte et al., 2016). Liu et al. provided the exact location by randomly selecting cytosine sites within the peak region detecting by MeRIP-seq (Liu Y. et al., 2020). However, such a strategy may lead to many false-positive samples (Kunqi Chen et al., 2019). To avoid such uncertainty, we proposed a model based on low-resolution data.

The rapid development of deep learning has promoted natural language processing studies. Word2vec is a remarkable achievement in natural language processing technology (Mikolov et al., 2013). Distributed representation of word vector is the core idea of word2vec, which means the representation of a word can be inferred from its context. With the development of high-throughput sequencing technology, the sequencing quality of biological sequences can be guaranteed. Therefore, some researchers in bioinformatics regard the biological sequences as a sentence, and k-mers as words. The word2vec method can then be applied to represent the biological sequences. Asgari et al. proposed BioVec based on the skip-gram model for biological sequences representation (Asgari and Mofrad, 2015). Kimothi et al. developed a model named seq2vec based on doc2vec, which is an extension of the original word2vec (Kimothi et al., 2016). The dna2vec model is dedicated to representing variable-length words (Ng, 2017a). It has been applied to several topics in bioinformatics. For example, Deng et al. proposed D2VCB for predicting protein–DNA-binding sites based on k-mer embeddings (Deng et al., 2019). Hong et al. applied the pretrained k-mer embeddings to encode enhancers and promoters (Hong et al., 2020). We employed the dna2vec embeddings to represent k-mers of *Drosophila* genomic sequences.

In this study, we represent the RNA sequences by using feature embeddings. We applied an SVM classifier to create a model for predicting 5hmC modification sites. Our model was trained on the low-resolution modification datasets, which is more reliable than the 1-base resolution set. The result suggests that our model is effective in identifying 5hmC sites.

## Materials and Methods

### Datasets

**FIGURE 1:**
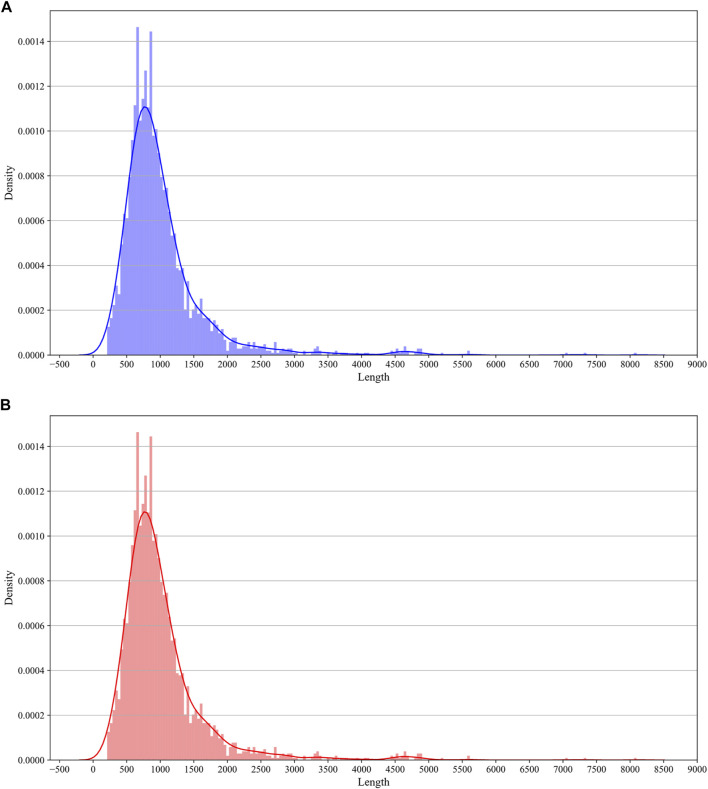
Sequence length distribution. The X-axis represents the length of sequences. The Y-axis represents the density of distribution. **(A)** Histogram density for the distribution of the length of positive sequences. **(B)** Histogram density for the distribution of the length of positive sequences.

In this study, we constructed the benchmarking dataset according to the experimental result from Delatte et al. (2016). The result from Delatte et al. contains 3058 peak regions distributed on chromosomes, which contain chr2L, chr2R, chr3L, chr3R, chr4, chrX, chr2RHet, chr3LHet, chr3RHet, chrYHet, chrU, and chrUextra. According to Hoskins et al. (2015), the genome sequences are of high quality on chr2L, chr2R, chr3L, chr3R, chr4, and chrX, while the remaining chromosome sequences are of low quality. Therefore, we only used the sequence data from chr2L, chr2R, chr3L, chr3R, chr4, and chrX. We got 2616 peak regions containing 5hmC modification sites. Subsequently, we obtained the transcription direction of every region by querying the UCSC genome browser tracks (Karolchik et al., 2003). Finally, 2616 positive samples were curated, which are regions containing 5hmC modification sites. Non-peak regions within transcripts carrying peak regions are curated as negative samples. The non-peak regions were cropped to the same lengths as the peak regions in a one-vs.-one strategy. A total of 2616 positive samples and 2616 negative samples were finally curated. We plot the density distribution of sequence lengths in Figure 1.

### *K*-Mer Embeddings

*K*-mer is a common and efficient way to represent RNA sequences, which divided the biological sequences into short segments of the length *k*. We employed the *k*-mer embeddings for representing the *k*-mer instead of one-hot encoding. *K*-mer embeddings can capture semantic and linguistic analogies and avoid the curse of dimensionality (Mikolov et al., 2013). The dna2vec model was used in this study for training *k*-mer embeddings (Ng, 2017a, 2). The corpus was collected from dm3 (Karolchik et al., 2003) genome assembly. We selected high-quality six chromosome sequences from dm3, including ch2L, chr2R, chr3L, chr3R, chr4, and chrX. The corpus was used as the input of the dna2vec. *K*-mer embeddings were obtained by training dna2vec. Let *p* (*k*, *i*) (*i* = 1, 2,…4^*k*^) represent the *i*-th type *k*-mer fragment. The process of the dna2vec model can be expressed as follows:

$$p\left(k,i\right)\overset{h\left(\cdot \right)}{\to }v\left(p\left(k,i\right)\right),$$ (1)

where *h*(.) is the mapping from a *k*-mer fragment to *k*-mer embedding and **v**(*p* (*k*, *i*)) is the embedding vector of the *i*-th type of *k*-mer. In this study, we chose *k* from 3 to 8. The dimension of **v**(*p* (*k*, *i*)) was set to 100.

### Distribution Representation of RNA Sequences

Given an RNA sequence *r* with length *l*, it can be represented as follows:

$$r=n_{1}n_{2}\cdots n_{l},$$ (2)

where *n_u_* (*u* = 1, 2,…, *l*) represents *u*-th nucleotide in RNA sequence. The RNA sequences are segmented into *k*-mers in an overlapping way. For example, we convert AUAGC into three 3-mers: “AUA,” “UAG,” “AGC.” Therefore, sequence *r* divided by *k* can be represented as follows:

$$r=\left\{w_{1},w_{2},\ldots ,w_{l-k+1}\right\},$$ (3)

where *w*_*j*_ (*j* = 1, 2,…, *l−k+1*) ∈ {*p*(*k*, *i*) |*k* = 3, 4,…, 8, *i* = 1, 2,…, 4^*k*^}. The fragment of *k*-mer RNA sequence can be considered as an RNA word. With the mapping *h*(.) from dna2vec, *w*_*i*_ was converted into the corresponding embedding vector. Sequence *r* can be expressed in a matrix as follows:

$$E\left(r,k\right)=\left[\begin{array}{cccc} v\left(w_{1}\right) & v\left(w_{2}\right) & \ldots & v\left(w_{l-k+1}\right) \end{array}\right],$$ (4)

Since dna2vec was trained by a corpus of DNA sequences, the *k-*mers from dna2vec do not contain uracil. We replaced thymine with uracil on *k*-mers for using the mapping. Considering the sum of dna2vec embeddings along the sequence is related to concatenating *k*-mers (Ng, 2017b), we sum the embedding vector in *E(r, k)* for representing the sequence *r*, as follows:

$$e\left(r,k\right)=\sum_{i=1}^{l-k+1}v\left(w_{i}\right)/l-k+1.$$ (5)

In this study, we chose *k* = 3, 4, 5, 6, 7, and 8. The final feature vector is formed by concatenating **e**(*r*, *k*) with different *k*, as follows:

$$e\left(r\right)={\left[\begin{array}{cccc} e{\left(r,3\right)}^{T} & e{\left(r,4\right)}^{T} & \ldots & e{\left(r,8\right)}^{T} \end{array}\right]}^{T}.$$ (6)

### Model Construction Algorithm

We evaluated three machine learning algorithms in this task, including SVM, CNN, and C4.5 classification tree. For the SVM classifier, we applied the radial basis function (RBF) kernel, as follows:

$$\kappa \left(e_{i},e_{j}\right)=\exp\left(-\gamma {\left\|e_{i}-e_{j}\right\|}^{2}\right),$$ (7)

where *γ* is a parameter and ||.|| vector norm operator.

For the CNN classifier, the max-pooling layer and dropout layer are used to avoid the over-fitting problem. The sigmoid function followed by a fully connected network is applied for performing the output. We used stochastic gradient descent to optimize parameters (Bottou, 2012). The binary cross-entropy function is used as the loss function (de Boer et al., 2005), as follows:

$$L\left(\theta \right)=\frac{1}{N}\sum_{i=1}^{N}y_{i}\log\left(h_{\theta }\left(e\right)\right)-\left(1-y_{i}\right)\log\left(\mathrm{1-}h_{\theta }\left(e\right)\right),$$ (8)

where *y_i_* is the label of the i-th sample, h_*θ*_(**e**) the output of the neural network, and N the number of samples.

For C4.5 algorithm, the information gain ratio for selecting appropriate features is defined as follows:

$$G_{r}\left(D,e_{i}\right)=\frac{G\left(D,e_{i}\right)}{IV\left(e_{i}\right)},$$ (9)

where *D* is the whole dataset, *G*_*r*_ (*D, e*_*i*_) the information gain, *IV*(*e*_*i*_) the intrinsic value of *e*_*i*_ (Salzberg, 1994), and *e*_*i*_ the *i*-th feature of feature **e**.

### Degree of Separation

To measure the degree of separation in the visualization analysis, we introduced the J-score. We first define the intra-class divergence sw and interclass divergence sb, as follows:

$$s_{b}=\left({\bar{e}}_{+}-{\bar{e}}_{-}\right){\left({\bar{e}}_{+}-{\bar{e}}_{-}\right)}^{T},$$ (10)

$$s_{w}=\sum_{j=1}^{m_{+}}\left(e_{+}\left(r_{j}\right)-{\bar{e}}_{+}\right){\left(e_{+}\left(r_{j}\right)-{\bar{e}}_{+}\right)}^{T}+\sum_{j=1}^{m_{-}}\left(e_{-}\left(r_{j}\right)-{\bar{e}}_{-}\right){\left(e_{-}\left(r_{j}\right)-{\bar{e}}_{-}\right)}^{T},$$ (11)

where

$${\bar{e}}_{+}=\frac{1}{m_{+}}\sum_{j=1}^{m_{+}}e_{+}\left(r_{j}\right),$$ (12)

$${\bar{e}}_{-}=\frac{1}{m_{-}}\sum_{j=1}^{m_{-}}e_{-}\left(r_{j}\right),$$ (13)

where *e_+_(r_j_)* is the feature vector of the j-th positive sample, *e_-_(r_j_)* is the feature vector of the j-th negative sample, and m+ and m- are the number of positive and negative samples, respectively.

The J-score can now be defined as follows:

$$J=\frac{s_{b}}{s_{w}}.$$ (14)

The higher J-score indicates a better degree of separation between positives and negatives.

### Framework of This Study

**FIGURE 2:**
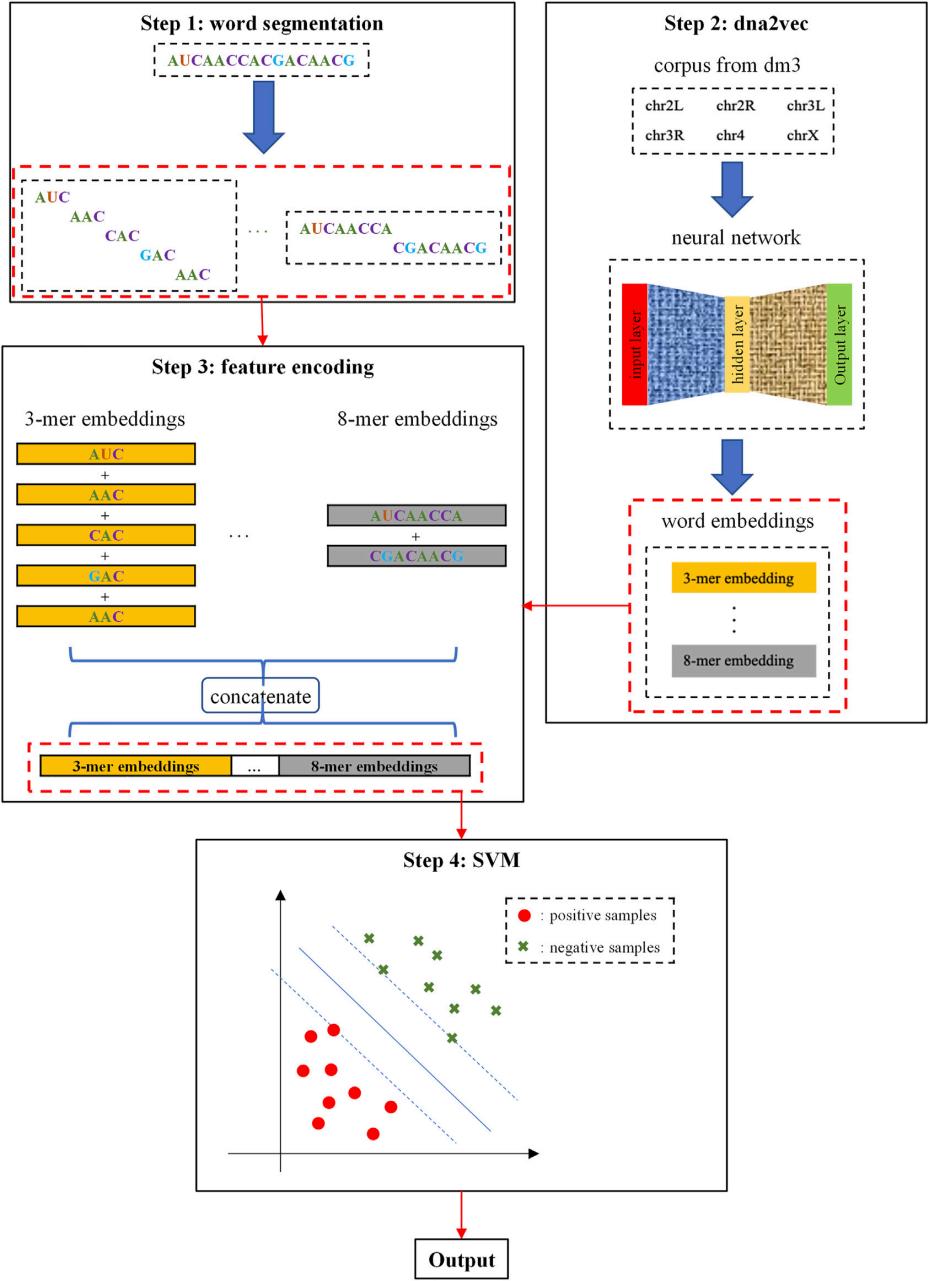
Flowchart of this study. Step 1: RNA sequences are segmented into *k*-mers in the overlapping way, where *k* = 3, 4, 5, 6, 7, 8. Step 2: *k*-mers embeddings were trained by the dna2vec model with corpus from dm3. Step 3: We perform summation and concatenation on these *k*-mers embeddings to encode RNA sequences. Step 4: SVM is used as a classifier for distinguishing positive and negative samples.

The framework of i5hmcVec is illustrated in Figure 2. We obtained the *k*-mer embeddings using dna2vec (Ng, 2017a), which is trained by the *Drosophila* genome sequences version dm3. RNA sequences were encoded by the embedding vectors for variable-length *k*-mers. SVM was applied as a classifier to distinguish the positive and negative samples.

### Parameter Calibration

In this section, we give a detailed introduction to optimizing parameters. SVM was implemented by the Python package scikit-learn. We chose to use the radial basis function (RBF) as the kernel function. A grid search strategy was applied to find the optimal parameters *c* and *γ*. The parameter *c* is the cost parameter in SVM, while *γ* is the parameter in the RBF kernel function. The range of parameter c is (2^-5^, 2^15^), while the range for parameter *γ* is (2^-15^, 2^-5^). The step for generating the logarithm searching grid is 2 and 2-1 for c and *γ*, respectively. The CNN algorithm is implemented by Keras. The batch size was set to 16. A logarithm grid search strategy was used to find the optimal parameters epoch e and learning rate a. The range of parameter a: 10^-4^, 5 × 10^-4^, 10^-3^, 5 × 10^-3^, 10^-2^, and 5 × 10^-2^. The range of parameters e is 100, 150, 200, 250, and 300. We used the weka package to implement C4.5. We evaluated the performance on different parameters C, which is the confidence threshold for pruning. The range of C is [0.2, 0.5] with a step of 0.05.

### Performance Measures

Four statistics, including sensitivity (Sen), specificity (Spe), accuracy (Acc), and Matthews correlation coefficient (MCC), were used to measure the prediction performance of our method. These performance measures can be defined as follows:

$$Sen=\frac{TP}{TP+FN},$$ (15)

$$Spe=\frac{TN}{TN+FP},$$ (16)

$$Acc=\frac{TP+TN}{TP+FP+TN+FN},\text{and}$$ (17)

$$MCC=\frac{TPTN-FPFN}{\sqrt{\left(TP+FN\right)\left(TN+FN\right)\left(TP+FP\right)\left(TN+FP\right)}},$$ (18)

where TP, TN, FP, and FN are the number of true positives, true negatives, false positives, and false negatives in the cross-validation process, respectively.

In addition, we also draw the receiver operating characteristic (ROC) curve and precision–recall (PR) curve to describe the performance of our method. The area under the ROC curve (AUROC) and the area under the PR (AUPR) curve were also recorded as performance indicators.

## Results

### Performance of Diffident Kind Features and Classifiers

In this study, nine kinds of *k*-mer embeddings were obtained, including six kinds of single *k* value embeddings and 3 kinds of multiple *k* value combinations. The single *k* values range from 3 to 8. The multiple *k* value combinations include the 4, 5, 6-mer combination, 6, 7, 8-mer combination, and 3, 4, 5, 6, 7, 8-mer combination. We first evaluate the performance of each single *k* value embedding. After that, we evaluate three multiple *k* value combinations.

**FIGURE 3:**
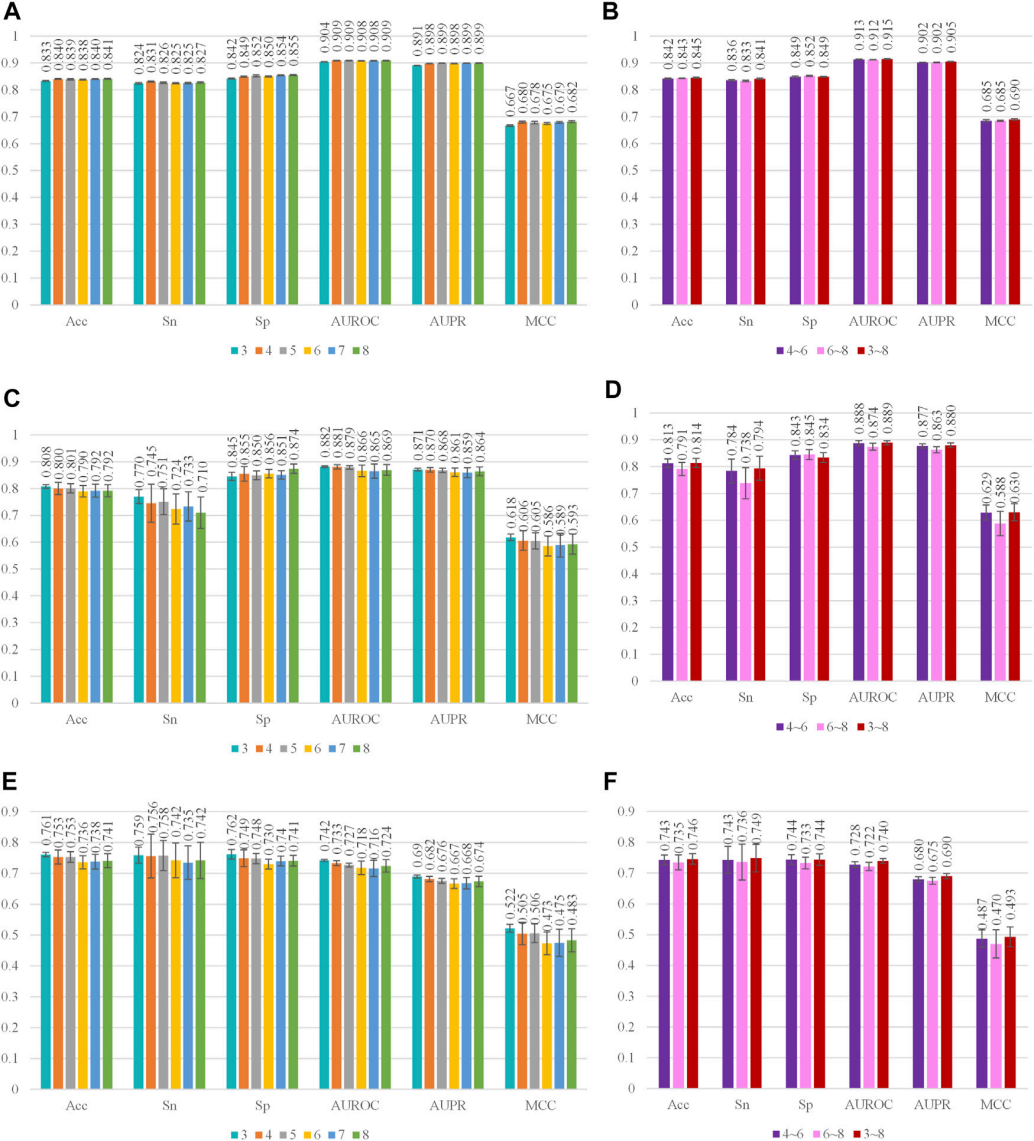
Performance of different kinds features on SVM, CNN, and C4.5. Cyan, orange, gray, yellow, blue, and green, respectively, represent the performance of 3-mer, 4-mer, 5-mer, 6-mer, 7-mer, and 8-mer embedding features. Purple, pink, and red, respectively, represent the performance of 4, 5, 6-mer concatenated embeddings, 6, 7, 8-mer concatenated embeddings, and 3, 4, 5, 6, 7, 8-mer concatenated embeddings. **(A,B)** Performance of different kinds of feature on SVM. The standard deviation of SVM on 3-mer, 4-mer, 5-mer, 6-mer, 7-mer, 8-mer, 4, 5, 6-mer, 6, 7, 8-mer, and 3, 4, 5, 6, 7, 8-mer is in the range (0.001, 0.003), (0.001, 0.003), (0.001, 0.003), (0.001, 0.003), (0.001, 0.003), (0.001, 0.004), (0.001, 0.004), (0.001, 0.003), and (0.001, 0.003); **(C,D)** Performance of different kinds of feature on CNN. The standard deviation of CNN on 3-mer, 4-mer, 5-mer, 6-mer, 7-mer, 8-mer, 4, 5, 6-mer, 6, 7, 8-mer, and 3, 4, 5, 6, 7, 8-mer is in the range (0.003, 0.026), (0.008, 0.071), (0.006, 0.049), (0.015, 0.056), (0.016, 0.055), (0.016, 0.059), (0.008, 0.044), (0.011, 0.058), and (0.008, 0.045); **(E,F)** Performance of different kinds of features on C4.5. The standard deviation of CNN on 3-mer, 4-mer, 5-mer, 6-mer, 7-mer, 8-mer, 4, 5, 6-mer, 6, 7, 8-mer, and 3, 4, 5, 6, 7, 8-mer is in the range (0.005, 0.545), (0.007, 0.531), (0.005, 0.049), (0.007, 0.685), (0.003, 0.440), (0.007, 0.489), (0.008, 0.630), (0.006, 0.567), and (0.005, 0.518).

Three machine learning-based classifiers were applied in this study. They are SVM, CNN, and C4.5. The parameters of these classifiers are optimized as in the method section. The optimization process is recorded as mesh surf plots in Supplementary Figures S1–S3 in the supplementary materials. The data for quantitative analysis is recorded in Supplementary Tables S1–S27. The optimal parameters for different classifiers are: the c and *γ* of SVM on the 3-mer, 4-mer, 5-mer, 6-mer, 7-mer, 8-mer, 4, 5, 6-mer, 6, 7, 8-mer and 3, 4, 5, 6, 7, 8-mer are (29, 2–5), (27, 2–5), (27, 2–5), (27, 2–5), (27, 2–5), (27, 2–5), (25, 2–5), (25, 2–5), and (24, 2–5); the a and e of CNN on the 3-mer, 4-mer, 5-mer, 6-mer, 7-mer, 8-mer, 4, 5, 6-mer, 6, 7, 8-mer and 3, 4, 5, 6, 7, 8-mer are (5 × 10^-2^, 200), (5 × 10^-2^, 150), (5 × 10^-2^, 150), (5 × 10^-2^, 250), (5 × 10^-2^, 150), (5 × 10^-2^, 250), (5 × 10^-2^, 150), (5 × 10^-2^, 100), and (5 × 10^-2^, 150); the C of C4.5 on the 3-mer, 4-mer, 5-mer, 6-mer, 7-mer, 8-mer, 4, 5, 6-mer, 6, 7, 8-mer and 3, 4, 5, 6, 7, 8-mer are 0.45, 0.3, 0.2, 0.5, 0.2, 0.45, 0.25, 0.3, and 0.3. The performances of all models are evaluated by 10 times 5-fold cross-validations. The optimal performance is recorded in Figure 3 and Supplementary Tables S28–S54.

### Semantic Symmetry of *K*-Mer Embeddings

**FIGURE 4:**
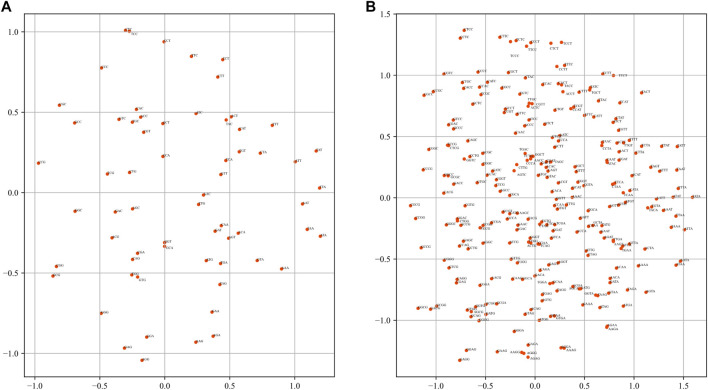
Visualization of *k*-mer embeddings with PCA. Each dot represents a *k*-mer embedding vector. **(A)** 3-mer embedding. **(B)** 4-mer embedding.

One of the most important functions of word2vec is that the word embeddings can solve semantic and linguistic analogies (Mikolov et al., 2013). Therefore, the semantic relation of the *k*-mer embeddings from dna2vec needs to be discussed. Principal component analysis (PCA) was applied to reveal the relationship of *k*-mer fragments. For 5-mer embeddings, the number of words is 1024. To present the results clearly, we only plot the PCA results of 3-mer and 4-mer embeddings in Figure 4. As in Figure 4, many words show symmetry trends about the horizontal axis, such as (CGC, GCG), (CTT, AAG), and (TACT, AGTA). Many words with such property have the characteristics of complement or reverse complement. Zou et al. regarded this phenomenon as semantic symmetric in the human genome (Zou et al., 2019). We observe and confirm this phenomenon in *Drosophila* genome.

### Feature Visualization

**FIGURE 5:**
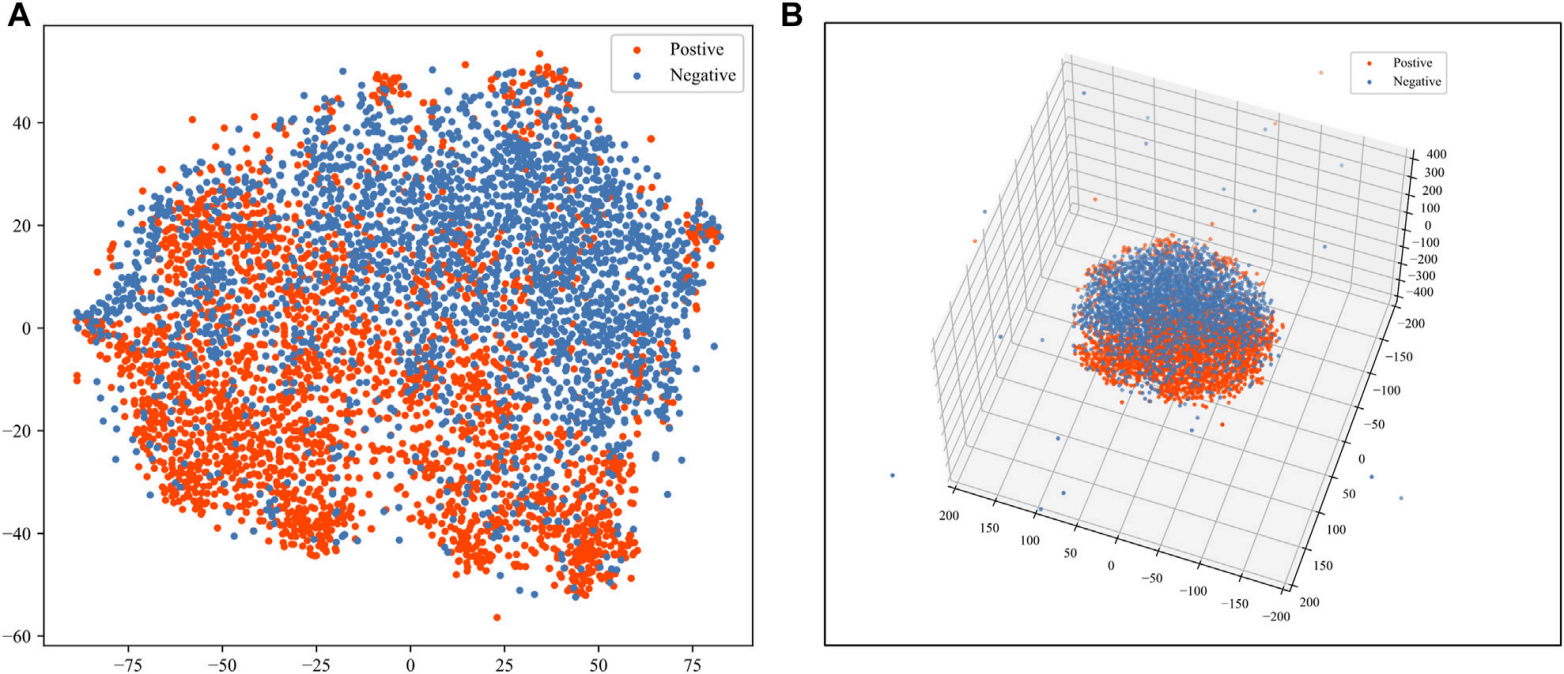
Visualization of sequence features. The red dots represent the positive samples. The blue dots represent the negative samples. **(A)** Visualization of 2-dimensional t-SNE. **(B)** Visualization of 3-dimensional t-SNE.

We used the t-distributed stochastic neighbor embedding (t-SNE) (van der Maaten and Hinton, 2008) method to help visualize the sequence features. The t-SNE algorithm is an effective way of reducing dimensions for visualization purposes. According to the visualization of t-SNE, we can judge whether the positive and negative samples are separable in the feature space. We applied the t-SNE for reducing the dimension of the feature to 2 and 3. We also calculated the J-score, which has been elaborated in the method section, as a quantitative separation measure in the reduced feature space. As shown in Figure 5, positive and negative samples are highly separable. The J-score of 2 and 3 dimensions of t-SNE are 0.202 and 0.165, indicating an acceptable level of separation.

### Performance Comparison With Existing Methods

The i5hmCVec is constructed based on a low-resolution modification dataset. WeakRM (Huang et al., 2021) was also proposed for identifying the 5hmC modification sites on low-resolution data. We summarized the dataset distribution used in the i5hmCVec and WeakRM in Table 1.

**TABLE 1:** Dataset distributions of i5hmcVec and WeakRM.

Method	Positive^a^	Negative^b^	Window size
i5hmCVec	2616	2616	209 nt∼8097 nt
WeakRM (training)	1875	1875	210 nt∼8090 nt
WeakRM (validation)	235	235	210 nt∼8090 nt
WeakRM (testing)	234	234	210 nt∼8090 nt

a Positive samples are sequences, which contain the 5hmC sites.

b Negative samples are sequences, which do not contain the 5hmC sites.

**FIGURE 6:**
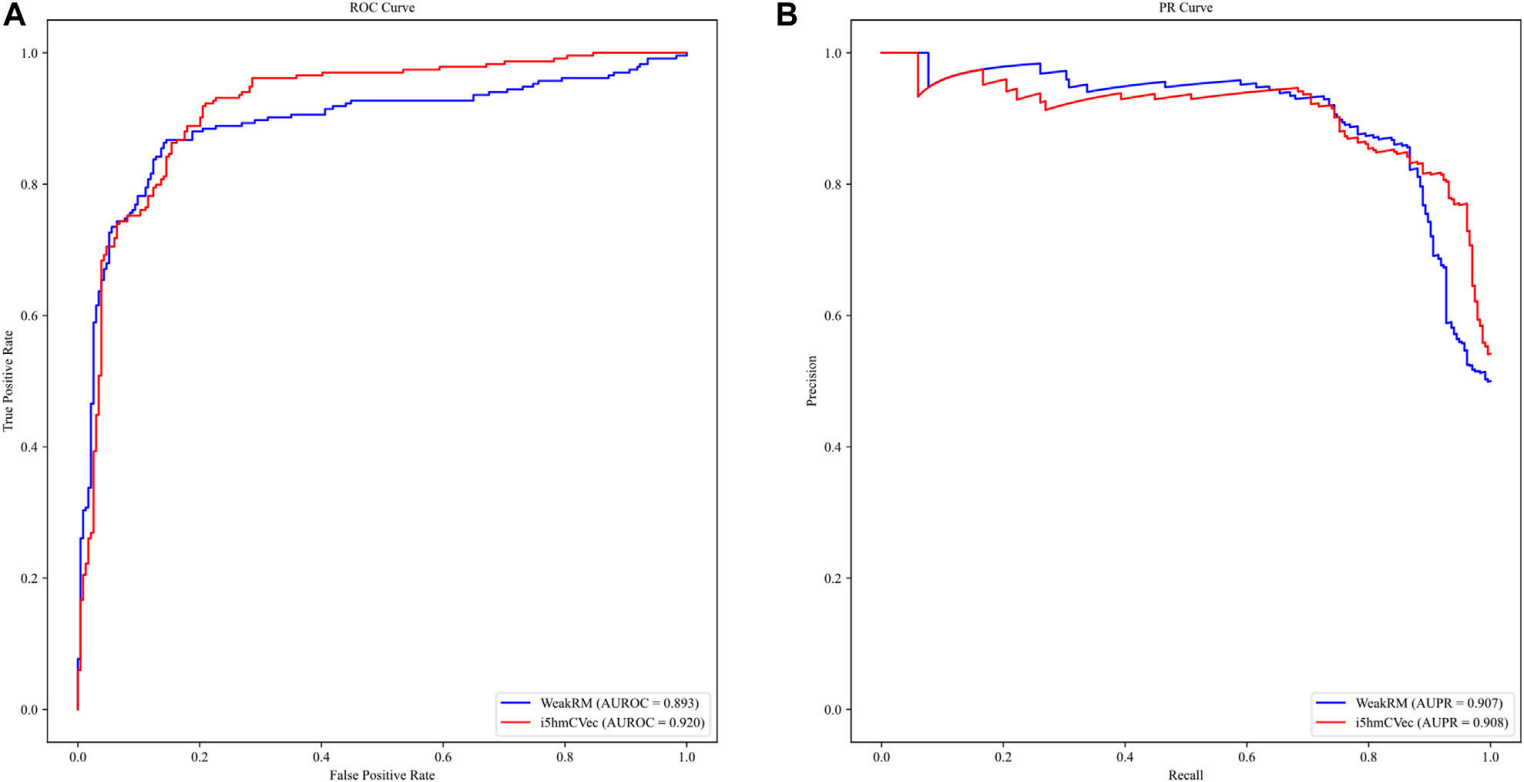
ROC and PR curves of i5hmcVec and WeakRM on the dataset from WeakRM. **(A)** ROC curve. The X-axis is the false positive rate, and the Y-axis is the true positive rate. **(B)** PR curve. The X-axis is the recall, and the Y-axis is the precision.

We used the dataset from WeakRM for training the i5hmCVec model. We also reproduced WeakRM for obtaining more types of performance metrics. Due to inevitable randomness errors, our reproduced performances are slightly different from the original reports. The differences are so tiny that the comparison results would not change. As in Table 2, i5hmCVec achieved 0.846, 0.920, 0.908, and 0.692 on Acc, AUROC, AUPR, and MCC, respectively, which are higher than the performance values of WeakRM. In addition, we make a comparison of training time between i5hmcVec and WeakRM. Training WeakRM takes about 500 s, while i5hmCVec takes about 25 s. To describe the results more intuitively, we displayed the ROC curve and PR curve of two models, as in Figure 6. As in Figure 6, both the AUROC and AUPR of i5hmCVec are slightly better than the WeakRM. In total, iRNA5hmCVec achieved better performances than WeakRM on a low-resolution modification dataset.

**TABLE 2:** Performance of i5hmcVec and WeakRM on the dataset from WeakRM.

Method	Acc^a^	Sen^b^	Spe^c^	AUROR^d^	AUPR^e^	MCC^f^
WeakRM	0.790	0.617	**0.967**	0.892	0.905	0.619
i5hmCVec	**0.846** ^g^	**0.838**	0.855	**0.920**	**0.908**	**0.692**

a *Acc* is short for accuracy.

b *Sen* is short for sensitivity.

c *Spe* is short for specificity.

d AUROC means the area under the ROC curve.

e AUPR means the area under the PR curve.

f *MCC* is short for Matthews correlation coefficient.

g Boldface indicates the best performance on each metric among methods.

## Discussion

Identifying modification sites is an important work for studying 5hmC modification. In this study, we used machine learning methods to construct the model. There are three key steps for a machine learning problem.

First, a high-quality dataset is essential for building an effective model. We constructed the low-resolution benchmarking dataset from experimental results (Delatte et al., 2016). We did not use the strategy of randomly selecting cytosine sites within peak regions like Liu Y. et al. (2020). Because such a strategy may lead to many false-positive samples (Kunqi Chen et al., 2019). In addition, to ensure high quality of sequences, we only employed the high-quality chromosomes sequences in the genome assembly.

Second, the samples from the dataset should be represented by an informative digital vector. We encode RNA sequences using the k-mer embeddings, which are derived from dna2vec. According to our results, the feature vector can effectively separate positive and negative samples. These results suggest that this encoding scheme is suitable for our study.

Finally, a suitable classifier should be used for constructing the model. We compared the performance of SVM, C4.5, and CNN. The SVM classifier has the best performance. In addition, we optimize the parameters using a grid search strategy.

Although our model was trained on low-resolution data, we tried to evaluate the performance of our model on high-resolution data. We performed 10 times 5-fold cross-validations on the benchmarking dataset from iRNA5hmC (Liu Y. et al., 2020). The sequence data in iRNA5hmC are 41 nt. The results are recorded in Table 3. According to the results, the i5hmCVec does not receive expected performance on a high-resolution modification dataset. We speculated that there may be two reasons for this phenomenon. One is the low quality of the high-resolution dataset. The high-resolution dataset of 5hmC modification was developed by Liu et al. with a random site picking strategy (Liu Y. et al., 2020), which may lead to many false positives.

**TABLE 3:** Performance of i5hmcVec and iRNA5hmC on the benchmark dataset from iRNA5hmC.

Method	Acc^a^	Sen^b^	Spe^c^	AUROC^d^	AUPR^e^	MCC^f^
iRNA5hmC	**0.655** **^g^**	**0.677**	0.644	**0.697**	**0.685**	**0.310**
i5hmcVec^h^	0.642	0.636	**0.647**	0.684	0.676	0.284
	±0.008	±0.010	**±0.009**	±0.007	±0.007	±0.016

a *Acc* is short for accuracy.

b *Sen* is short for sensitivity.

c *Spe* is short for specificity.

d AUROC means the area under the ROC curve.

e AUPR means the area under the PR curve.

f *MCC* is short for Matthews correlation coefficient.

g Boldface indicates the best performance on each metric among different methods.

h Performance of i5hmcVec on the benchmark dataset from iRNA5hmC with 10 times 5-fold cross-validation. Results are expressed as the mean and standard deviation of 10 times experiments.

**FIGURE 7:**
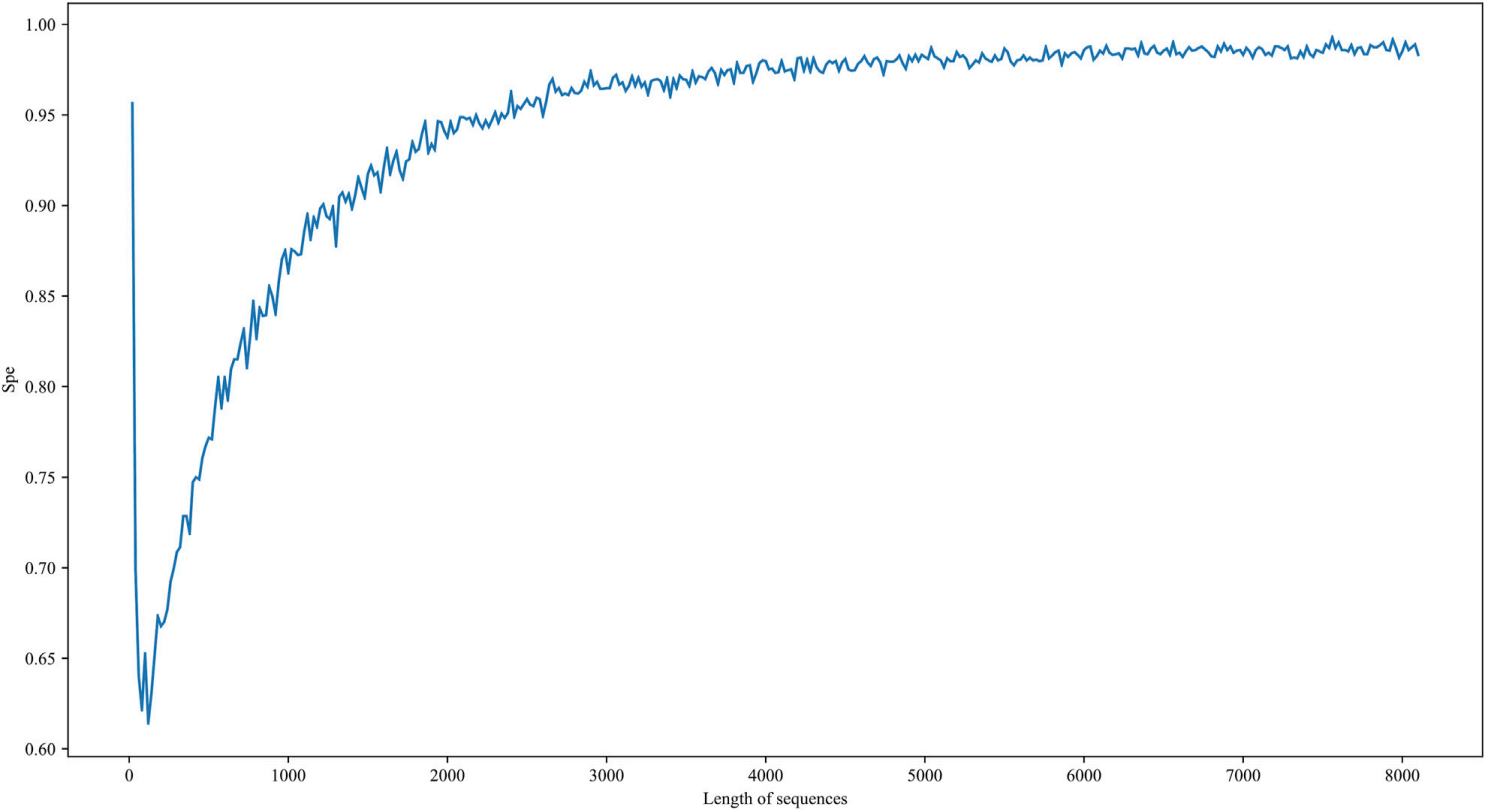
Performance of i5hmCVec on the negative datasets of different sequence lengths with an independent test. The X-axis is the length of sequences in the negative dataset. The Y-axis is the performance of i5hmCVec on spe.

The other is the limitation of resolution in our model. The length of low-resolution sequences is between 209 nt and 8097 nt, while the length of high-resolution sequences is 41 nt, which is much shorter than the lower bound of the low-resolution dataset. To estimate the resolution of our model, we evaluate the performance of the 5hmC on negative samples with different length restrictions. We re-select RNA sequences with sequence lengths ranging from 20 to 8100 on the non-peak region within the transcript carrying peak region as an independent testing dataset. It is worth noting that to prevent information leakage, there is no regional intersection between these negative samples and the negative samples in the benchmarking dataset. In addition, since there are only labels for negative samples, Spe is used as a performance metric. As shown in Figure 7, when the length of the sequence is less than 1000 nt, the performance of spe gradually drops. When the sequence length is around 100, the performance value takes a deep dive. Although the performance increases drastically when the sequence length is less than 100, we believe this is caused by over-fittings on negative samples. Therefore, the i5hmCVec model is not suitable for working on the high-resolution dataset.

## Conclusion

In this study, we proposed a novel model named i5hmCVec for identifying 5hmC modification sites. We proposed a high-quality low-resolution 5hmC modification dataset. We construct the i5hmCVec based on dna2vec technology. The i5hmCvec achieved better performances than state-of-the-art methods on a low-resolution dataset. In addition, we analyze the semantic symmetric with the *Drosophila* genome. We hope our findings may be useful for future studies.

## Data Availability

Publicly available datasets were analyzed in this study. These data can be found at: https://github.com/liu-h-y/5hmC_model.
