# Supplementary figures and images for: i5hmCVec: Identifying 5-Hydroxymethylcytosine Sites of Drosophila RNA Using Sequence Feature Embeddings
Source: Front Genet. 2022 May 3;13:896925. doi: 10.3389/fgene.2022.896925 (PMC9110757; doi:10.3389/fgene.2022.896925)

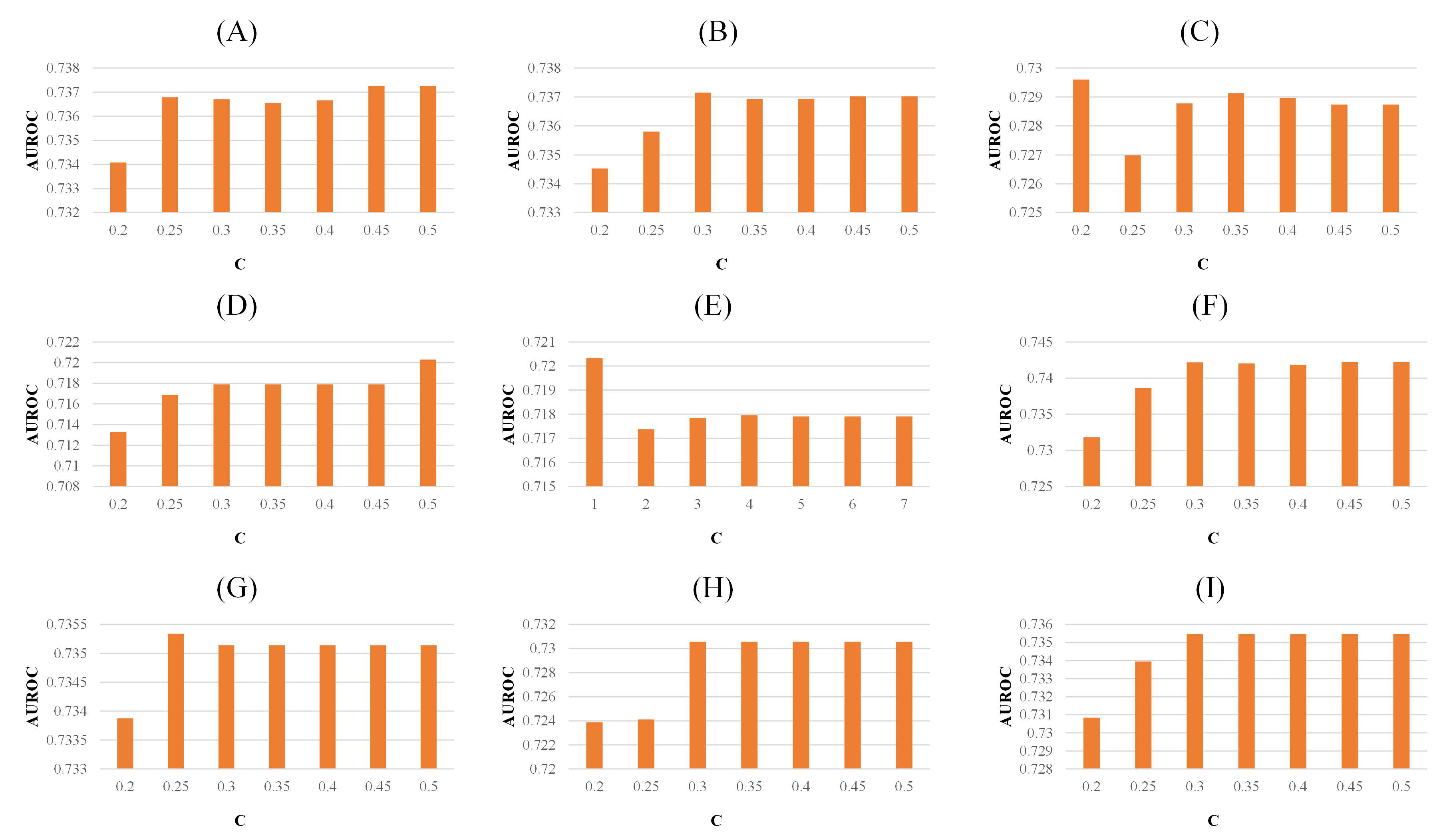

Supplement: Supplementary file 1 [file Image3.TIF]

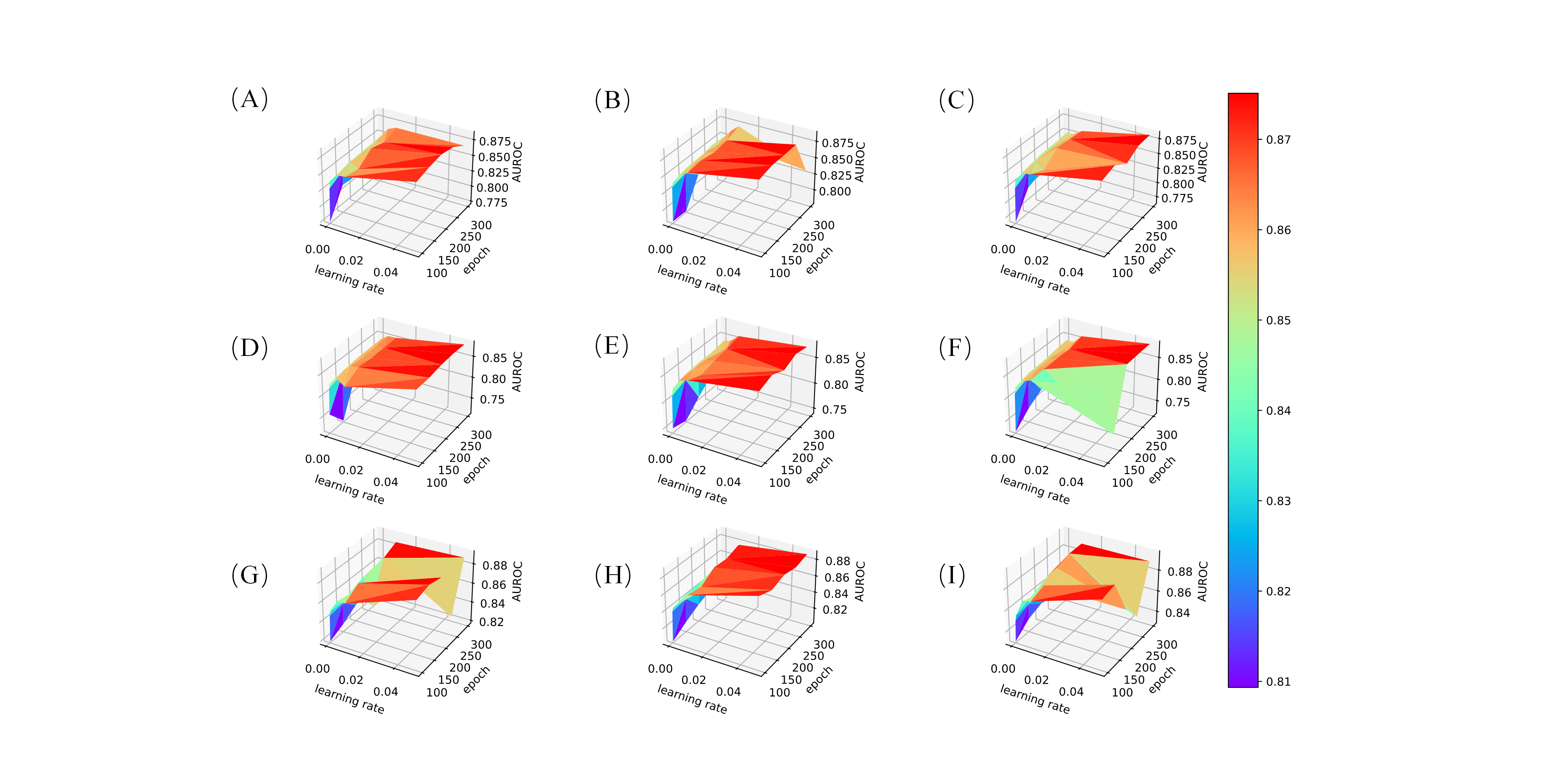

Supplement: Supplementary file 2 [file Image2.TIF]

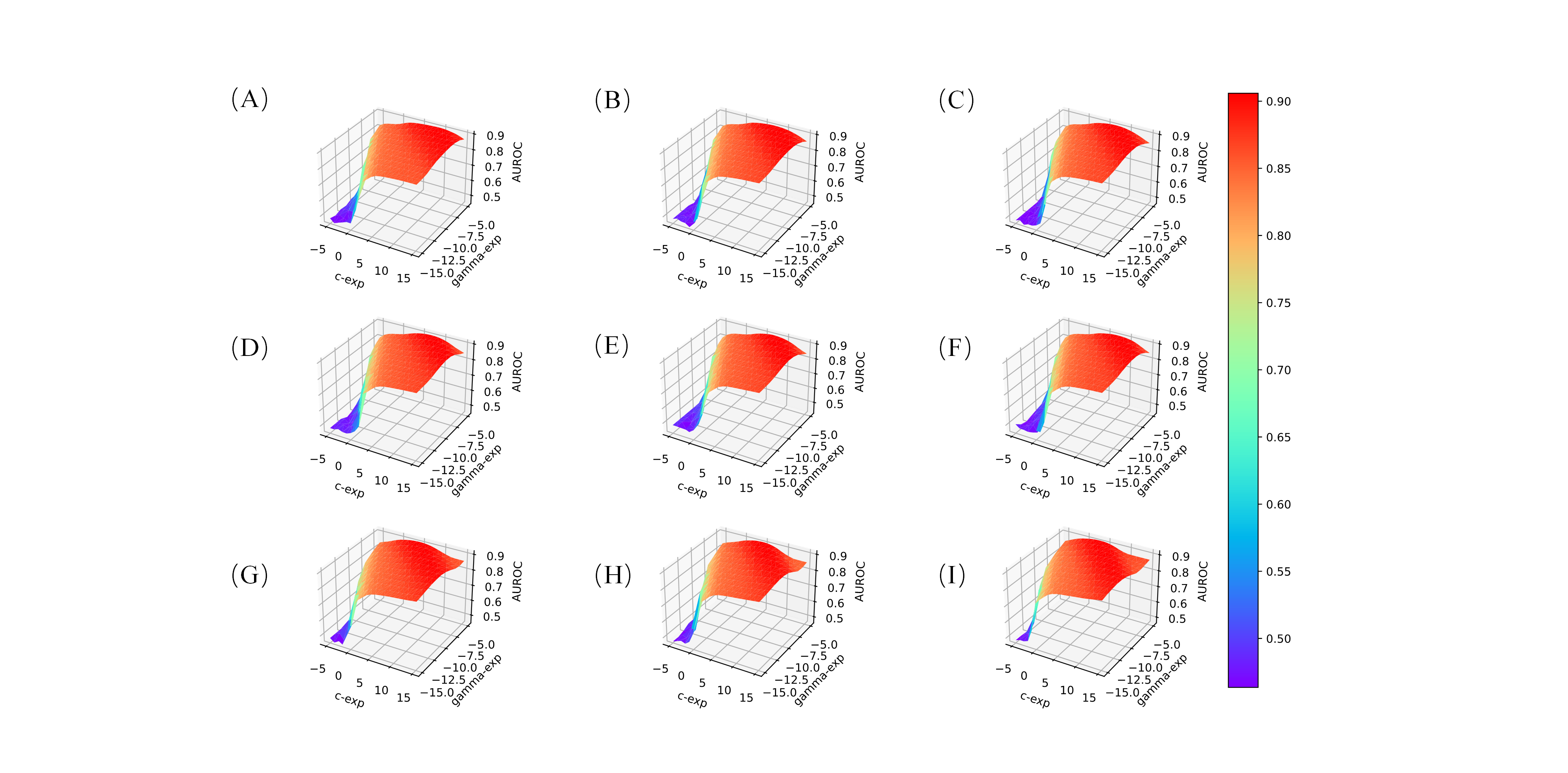

Supplement: Supplementary file 3 [file Image1.TIF]
