# Supplementary material for: i5hmCVec: Identifying 5-Hydroxymethylcytosine Sites of Drosophila RNA Using Sequence Feature Embeddings
Source: Front Genet. 2022 May 3;13:896925. doi: 10.3389/fgene.2022.896925 (PMC9110757; doi:10.3389/fgene.2022.896925)
Supplement: Supplementary file 4 [file Table2.DOCX]

**Supporting Information**

**Supplementary Figures**

**Figure S1.** Parameter tuning process of support vector machine (SVM). There are two optimized parameters *c* and *γ* in SVM model. The parameter *c* is the cost parameter in SVM, while *γ* is the parameter in the RBF kernel function. The X-axis c-exp denotes logarithm to base 2 of *c.* The Y-axis gamma-exp denotes logarithm to base 2 of *γ.* The Z-axis AUROC denotes the performance AUROC of SVM*.* We conduct experiments on 9 kinds of feature combinations. (A)The 3-mer embedding feature; (B)The 4-mer embedding feature; (C)The 5-mer embedding feature; (D)The 6-mer embedding feature; (E)The 7-mer embedding feature; (F)The 8-mer embedding feature; (G)The concatenating of 4, 5, 6-mer embedding feature; (H)The concatenating of 6, 7, 8-mer embedding feature; (I)The concatenating of 3, 4, 5, 6, 7, 8-mer embedding feature.

**Figure S2.** Parameter tuning process of convolutional neural network (CNN). There are two optimized parameters epoch *e* and learning rate *a* in CNN model. The X-axis is learning rate*.* The Y-axis is epoch*.* The Z-axis AUROC denotes the performance AUROC of CNN*.* We conduct experiments on 9 kinds of feature combinations. (A)The 3-mer embedding feature; (B)The 4-mer embedding feature; (C)The 5-mer embedding feature; (D)The 6-mer embedding feature; (E)The 7-mer embedding feature; (F)The 8-mer embedding feature; (G)The concatenating of 4, 5, 6-mer embedding feature; (H)The concatenating of 6, 7, 8-mer embedding feature; (I)The concatenating of 3, 4, 5, 6, 7, 8-mer embedding feature.

**Figure S3.** Parameter tuning process of C4.5. There are one optimized parameter *C*, confidence threshold for pruning, in C4.5. The X-axis is *C.* The Y-axis AUROC denotes the performance AUROC of C4.5*.* We conduct experiments on 9 kinds of feature combinations. (A)The 3-mer embedding feature; (B)The 4-mer embedding feature; (C)The 5-mer embedding feature; (D)The 6-mer embedding feature; (E)The 7-mer embedding feature; (F)The 8-mer embedding feature; (G)The concatenating of 4, 5, 6-mer embedding feature; (H)The concatenating of 6, 7, 8-mer embedding feature; (I)The concatenating of 3, 4, 5, 6, 7, 8-mer embedding feature.
